# Subclinical Cognitive and Neuropsychiatric Correlates and Hippocampal Volume Features of Brain White Matter Hyperintensity in Healthy People

**DOI:** 10.3390/jpm10040172

**Published:** 2020-10-15

**Authors:** Gianfranco Spalletta, Mariangela Iorio, Daniela Vecchio, Federica Piras, Valentina Ciullo, Nerisa Banaj, Stefano L. Sensi, Walter Gianni, Francesca Assogna, Carlo Caltagirone, Fabrizio Piras

**Affiliations:** 1 Laboratory of Neuropsychiatry, Department of Clinical and Behavioral Neurology, IRCCS Santa Lucia Foundation, 00179 Rome, Italy; mariang.iorio@gmail.com (M.I.); d.vecchio@hsantalucia.it (D.V.); federica.piras@hsantalucia.it (F.P.); v.ciullo@hsantalucia.it (V.C.); n.banaj@hsantalucia.it (N.B.); f.assogna@hsantalucia.it (F.A.); c.caltagirone@hsantalucia.it (C.C.); 2 Division of Neuropsychiatry, Menninger Department of Psychiatry and Behavioral Sciences, Baylor College of Medicine, Houston, TX 77030, USA; 3 Molecular Neurology Unit, Center of Advanced Studies and Technology (CAST), G. d’Annunzio University of Chieti-Pescara, 66100 Chieti, Italy; ssensi@uci.edu; 4 Department of Psychology, Sapienza University of Rome, Policlinico Umberto I, 00161 Rome, Italy; 5 Department of Neuroscience, Imaging, and Clinical Sciences, University G. d’Annunzio of Chieti-Pescara, 66100 Chieti, Italy; 6 Institute for Mind Impairments and Neurological Disorders, University of California-Irvine, Irvine, CA 92697, USA; 7 II Division of Internal Medicine and Geriatrics, Sapienza University of Rome, Policlinico Umberto I, 00161 Rome, Italy; walgianni@hotmail.com

**Keywords:** white matter hyperintensities (WMH), elderly subjects, hippocampus, apathy, anxiety, small vessel disease, magnetic resonance imaging (MRI)

## Abstract

White matter hyperintensities (WMH) are associated with brain aging and behavioral symptoms as a possible consequence of disrupted white matter pathways. In this study, we investigated, in a cohort of asymptomatic subjects aged 50 to 80, the relationship between WMH, hippocampal atrophy, and subtle, preclinical cognitive and neuropsychiatric phenomenology. Thirty healthy subjects with WMH (WMH+) and thirty individuals without (WMH−) underwent comprehensive neuropsychological and neuropsychiatric evaluations and 3 Tesla Magnetic Resonance Imaging scan. The presence, degree of severity, and distribution of WMH were evaluated with a semi-automated algorithm. Volumetric analysis of hippocampal structure was performed through voxel-based morphometry. A multivariable logistic regression analysis indicated that phenomenology of subclinical apathy and anxiety was associated with the presence of WMH. ROI-based analyses showed a volume reduction in the right hippocampus of WMH+. In healthy individuals, WMH are associated with significant preclinical neuropsychiatric phenomenology, as well as hippocampal atrophy, which are considered as risk factors to develop cognitive impairment and dementia.

## 1. Introduction

The aging brain undergoes multiple structural and functional changes [1]. A significant sign of aging is the deterioration of the cerebral white matter (WM) [2], a phenomenon due to myelin breakdown [3], alteration of the neurovascular unit, and disruption of its microstructural organization [4,5,6]. The presence of white matter hyperintensities (WMH) [7] is a well-known neuroradiological sign of these age-related changes in WM. The alterations are appreciated with Magnetic Resonance Imaging (MRI) and appear as brain areas of increased signal intensity on T2-weighted (T2-w) or fluid-attenuated inversion-recovery (FLAIR) scans [7]. Evidence supports the view that WMH can be highly predictive of stroke, dementia, and death [8].

The clinical significance of WMH has been extensively investigated in the elderly. WMH adversely impact cognitive, motor, and affective/motivational characteristics [9], and are a common feature in patients with dementia [9,10,11,12,13]. They can also be found in healthy (mostly but not exclusively elderly) subjects not showing signs of cognitive impairment [14,15,16]. In particular, evidence shows that general cognitive and motor performance in independently living elderly correlates with WMH burden, and declines along with WMH progression [15,17,18]. Moreover, WMH have been associated, in cross-sectional and longitudinal studies, with defective processing speed and attention, executive dysfunctions and deficits in explicit memory [19], and progression of WMHs associated with worse cognitive functioning.

The link between WM changes and occurrence of behavioral symptoms is also a matter of investigation. Evidence indicates that increased WMH load is associated with neuropsychiatric phenomenology such as depression, apathy, and anergia [20,21,22,23]. However, while some studies suggested a significant association between WMH and depressive symptoms [24,25,26,27], others failed to confirm this link in the elderly [28,29,30]. One possible source of variation among studies lies in the difference in methodological approaches adopted. For instance, some studies addressed the presence and severity of WMH using visual rating scales (e.g., [24]), thus resulting in poor WMH scores variability, others employed more continuous, volumetric approaches (e.g., [29]). Moreover, difficulties in differentiating among symptoms of depressed mood, apathy, anhedonia, or anergia [31,32] may explain in part such contrasting results. Recent data suggested that apathy but not depression is associated with a widespread reduction in white matter integrity within cortical-subcortical networks related to emotion regulation, reward, and goal-directed behavior [33].

The pathophysiological process associated with neuropsychiatry phenomenology relies on the disruption of subcortical-cortical connections caused by changes in WM integrity, which can impact the cortical gray matter (GM) integrity and also lead to cognitive impairment [34]. Supporting this notion, WMH are often associated with hippocampal atrophy (HA), an MRI signature of ongoing cognitive impairment and/or dementia [9]. The hippocampus is vulnerable to vascular factors [35] and several studies reported an association between WMH burden and alterations of hippocampal volume [36,37,38,39,40,41]. However, the vast majority of studies investigated WMH and HA but not in association with clinical manifestations.

Here we aimed at clarifying the associations between WMH loads, HA and neuropsychological and neuropsychiatric symptoms in a cohort of healthy people aged between 50 and 80, an age range where the occurrence of WMH is probable. In order to overcome the limitations of previous studies, WMH have not been rated using visual qualitative or semi-quantitative scales, but rather quantified using a continuous measure (volume load) through a methodological approach developed by our research group [11], thus covering the whole spectrum of WMH presentation.

Given the role of WM integrity in the modulation of neuropsychiatric symptoms (even at a subthreshold level) [42], and cognitive functioning [43], we predict that subjects with WMH will show initial neuropsychiatric symptoms (e.g., subclinical apathy, depression, and anxiety), worse cognitive performance and hippocampal volume reduction.

## 2. Materials and Methods

### 2.1. Subjects

We included 30 healthy subjects with WMH (WMH+) and 30 healthy subjects without (WMH−). The presence of WMH was defined as at least 1 lesion greater than 150 mm^3^ in FLAIR MRI images [44] according to our semi-automated method [11]. The 30 WMH− had no lesions. The two groups were matched in terms of age, gender, and educational level one by one (±3 years for age and ±1 year for education) and recruited from the same geographical area. 

All subjects were originally recruited from community recreational centers and hospital personnel. Inclusion criteria were (i) age between 50 and 80 years (ii) suitability for MRI scanning. The presence of WMH was detected in MR images by an expert clinical neuroradiologist and by a senior researcher, expert in MRI visual analyses (FaPi). Exclusion criteria included: (i) suspected cognitive impairment based on a Mini-Mental State Examination (MMSE) score <24 [45], and confirmed through the administration of the Mental Deterioration Battery (MDB) [46]; (ii) subjective complaint of memory difficulties or any other cognitive deficits interfering with daily living activities; (iii) vision and hearing loss that could interfere with testing procedures; (iv) major medical illnesses; (v) current or lifetime history of DSM-5 mental and personality disorders (assessed by the SCID-5-RV and SCID-5-PD or neurological (assessed by a clinical neurological evaluation) disorders (e.g., Parkinson’s disease, seizure disorder, head injury with loss of consciousness or any other significant mental or neurological disorder); (vi) history of large vessel disease such as stroke or transient ischemic attack (TIA); (vii) known or suspected history of alcohol or drug dependence and abuse during lifetime; (viii) non-Italian language native speaker. MRI exclusions criteria included severe motion artifacts precluding MRI interpretation and incomplete brain coverage.

The study followed the guidelines of the Santa Lucia Foundation Institutional Ethical Committee and, following the Helsinki Declaration, each subject signed an informed consent form before enrolment.

### 2.2. Cognitive Assessment

Two trained neuropsychologists performed the neuropsychological examination and were blind to all the medical information at the time of assessment. All tests were administered upon a single session and in a fixed predetermined order. Neuropsychological testing was performed on the same day of MRI scans. Based on the evidence from cross-sectional and longitudinal studies of a strong association between the presence and progression of WMHs and decreased cognitive functioning, defective processing speed and attention, executive dysfunctions, deficits in explicit memory and in perception/construction [19], such cognitive domains were thoroughly examined. Neuropsychological tests were selected according to their accuracy in differentiating subjects showing signs of mild cognitive impairment due to vascular disease [47]. Global cognitive functioning was evaluated using the MMSE [45], a widely used screening comprising tests of orientation, attention, memory, language and visual-spatial skills and characterized by excellent accuracy in separating vascular mild cognitively impaired patients from controls [47]. Tests extracted from the Mental Deterioration Battery (MDB) [46], a reliable instrument for neuropsychological diagnosis and characterization of the dementia syndrome, were used to evaluate: short and long-term verbal memory performance, by the Immediate (RIR) and Delayed (RDR) recall of Rey’s 15 words; the Controlled Word Fluency Test (WF) from the MDB and the Semantic Fluency Test (SF) [48] assessed the phonological and semantic processes central to speech production, and the executive processes implied in word search and switching between subcategories; non-verbal logical-deductive reasoning was evaluated by the Raven’s 47 progressive colored matrices (RPM); constructional praxis by the Copying of Drawings (CD) and Copying of Drawings with Landmarks (CDL). Additionally, the Copy (CROCF) and Delayed (DROCF) recall of the Rey-Osterrieth Complex Figure [49] were used to appraise constructional praxis for complex material and long-term visuospatial memory; the Trail Making Test (TMT) part A and B [50], a short, easily administered test highly efficient in differentiating patients with and without brain damage [51], was used to assess processing speed (TMT-A) and task switching abilities (TMT-B) while the Double Barrage Test (DBT) assessed visual attention; as to explore response inhibition abilities, the Wisconsin Card Sorting Test (WCST) (Heaton, 1993) and the abbreviated version of the Stroop test (ST) [52] were employed; shifting and response inhibition difficulties were inferred from the number of preservative errors in the WCST (WCST-Pers Err) and the interference effect (-ST_i_-) in the ST. The immediate and delayed recall of verbal material, the TMT-A and B, and the Stroop tests demonstrated in previous studies [53] high sensitivity (from 70 to 82%) and specificity (from 68 to 79%) in differentiating vascular mild cognitively impaired patients from subjects with normal cognition.

### 2.3. Psychiatric Assessment

Given the reported association between lacunar volumes in the white matter and depressed mood, anhedonia, apathy, and anergia [22], and based on the assumption that the presence of subclinical neuropsychiatric symptoms in healthy individuals may constitute a risk factor for progression to a clinical picture, being associated with microstructural variations in brain grey and with matter [42], a thorough neuropsychiatric examination was administered. The Hamilton Depression Rating Scale [54], the most commonly used measure of depression, with excellent validity in several different populations, and the Beck depression inventory (BDI) assessing characteristic attitudes and symptoms severity, were used to detect depression; the Apathy Rating Scale (ARS) evaluated the degree of apathy, i.e., a state characterized by simultaneous diminution in the overt behavioral, cognitive, and emotional concomitants of goal-directed behavior usually resulting from brain-related pathology [55]; considering the relationship between brain structure and subclinical anxiety condition [56] the State and Trait Anxiety Inventory (STAY 1-STAY 2) [57] and the Hamilton Anxiety Rating Scale (HAMA) [58] were used to investigate anxiety levels about an event, and anxiety levels as a personal characteristic and the severity of anxiety symptoms, respectively. The State-Trait Anger Expression Inventory (STAXI) [59] was used as an index of anger intensity as an emotional state (State Anger), and of the individual disposition to experience angry feelings as a personality trait (Trait Anger). Acute and chronic fatigue was measured using the Fatigue Rating Scale [60] as to appraise potential symptoms of exhaustion characterizing the pre-frail and frail syndromes, which are predictors of disability and hospitalization in cohorts of elderly people [61]. The clinical validity of the FRS is supported by a population study of fatigue in the general population [62].

### 2.4. Image Acquisition and Processing

The 60 participants underwent the same MRI protocol, including 2D FLAIR and whole-brain high-resolution T1-w images, using a 3T Allegra MR imager (Siemens, Erlangen, Germany) with a standard quadrature head coil. All planar sequences acquisitions were obtained in the plane of the AC–PC line. Subjects were centered in the head coil. And their movements were restrained with pillows. 2D FLAIR images were obtained in the axial plain (TE = 109 ms, TR = 8500 ms, slice thickness = 5 mm, slices = 24, matrix = 188 × 256, phase FOV = 0.73). T1-w images were obtained in the sagittal plane using a modified driven equilibrium Fourier transform (MDEFT) [63] (TE = 2.4 ms, TR = 7.92 ms, flip angle = 15°, voxel size = 1 mm × 1 mm × 1 mm). An experienced neuroradiologist and a trained rater (FaPi) inspected all the images to determine whether WMH were present and to differentiate WMH from acute stroke lesions or other brain abnormalities.

### 2.5. Regional Distribution and Volume Measurement of WMH 

WMH were detected in each FLAIR and T1-weighted images and measured with a semi-automated procedure, using a set of custom-made algorithms and software previously described [11]. To generate WMH maps, all the selected FLAIR and T1-w images were processed using the following steps:

(1) Preprocessing: (i) skull-stripping of the FLAIR images to restrict our analyses to brain tissue only, (ii) spatial normalization from native to stereotaxic space of the Montreal Neurological Institute (MNI) of the T1-weighted images. The resulting deformation parameters were then applied to FLAIR images for later statistical group comparisons and/or voxel-based statistics in a common coordinates system, (iii) removal of cerebellum and brainstem to exclude them from the analysis as WMH are rare in this brain regions, (iv) smoothing of skull-stripped FLAIR images with a 2 mm Full-Width Half Maximum (FWHM) Gaussian kernel; (2) Automated detection of WMH based on the intensity histogram of pre-processed FLAIR images. For each subject’s FLAIR images, we calculated the mean and standard deviation (SD) of the intensity of all brain voxels and applied a threshold value (the intensity mean +1.5 SD) to isolate probable WMH. Generated WMH maps were visually inspected and, if needed, manually corrected to exclude false classification of WMH from the map (needed in 5 images only, 16.6%); (3) Post-processing: generation of WMH map and volumetric estimation of the WMH load (expressed in mm^3^) at the individual and sample levels. WMH maps generated were binary images, and values assigned to each voxel (1–0) indicated the presence or absence of WMH. WMH load was calculated as the number of 1-coded voxels.

### 2.6. Hippocampal Volume Reduction

T1-w images were processed and examined using the VBM8 toolbox (available online: http://dbm.neuro.uni-jena.de/wordpress/vbm/ (accessed on 13 October, 2020)) implemented in the SPM8 software (Statistical parametric mapping software, SPM; Wellcome Trust Centre for Neuroimaging, UCL, London, UK; (available on line: https://www.fil.ion.ucl.ac.uk/spm/ (accessed on 13 October 2020)) running in Matlab 2007b (MathWorks, Natick, MA, USA).

The toolbox extends the unified segmentation model [64] consisting of MRI field intensity inhomogeneity correction, spatial normalization, and tissue segmentation at several pre-processing steps to further improve data quality. Initially, an optimized blockwise nonlocal-means filter [65] was applied to the images using the Rician noise adaption [66], in order to increase the signal-to-noise ratio. Subsequently, an adaptive maximum a posteriori segmentation approach extended by partial volume estimation [67] was employed to segment the T1-weighted images into GM, WM, and cerebrospinal fluid (CSF). The segmentation step was ended by applying a spatial constraint to the segmented tissue probability maps based on a hidden Markow Random Field model [68]. Then, the iterative high-dimensional normalization approach provided by the Diffeomorphic Anatomical Registration Through Exponentiated Lie Algebra (DARTEL) [69,70,71] toolbox was applied to the segmented tissue maps to register them to the MNI space. The tissue deformations were used to modulate participants’ GM maps to be entered in the analyses. Finally, the modulated and normalized GM segments were written with an isotropic voxel resolution of 1.5 × 1.5 × 1.5 mm and smoothed with a 6 mm Full-Width Half Maximum (FWHM) Gaussian kernel. The segmented, normalized, modulated and smoothed GM images were used for analyses and were focused on the hippocampus.

### 2.7. Statistical Analysis

#### 2.7.1. Neuropsychological and Neuropsychiatric Data

Demographic data were compared between groups using unpaired Student *t*-tests for age, educational attainment and general cognitive status as measured using the MMSE (see Table 1).

The effect of WMH on neuropsychiatric phenomenology was evaluated as follows. First, to estimate the odds of predicting the presence of WMH, separate binomial logistic regressions were conducted on group membership (WMH+ and WMH− as the dependent variable) considering each neuropsychological and neuropsychiatric variable as independent. Second, in order to test basic assumptions for a multivariable logistic regression model, the absence of multicollinearity, and the linearity in the logit for any continuous independent variables were examined. We checked, separately for the two groups, the tolerance value of each neuropsychological and neuropsychiatric predictor, that is the proportion of variation in each predictor independent from the correlation among regressors [72]. The tolerance value was calculated as 1-Rj^2^ where Rj^2^ is the coefficient of determination obtained by modeling the jth regressor (each neuropsychological test score and each neuropsychiatric variable that resulted as significant predictors in the univariate binomial logistic regressions) as a linear function of the remaining independent variables. The cut-off value was set such that the variability in a predictor, not related to other variables in the model, was at least 30%. In order to test the linearity of the relationships between the continuous predictors and their logit odds, the cross-products of each independent variable times its natural logarithm ([(X)ln(X)]) were included in two separate multivariable logistic regression models with the significant neuropsychological and neuropsychiatric predictors (as independent variables) and group membership as the dependent variable, and checked for significance [73]. If any of these terms was statistically significant (as indicated by a Wald Chi-Squared Test *p* < 0.05) the model was considered not accurate. These statistical tests allowed us to calculate and analyze the adjusted odds of predicting the presence of WHM for each neuropsychological and neuropsychiatric measure. 

#### 2.7.2. Neuroimaging–ROI analysis: Differences between Groups

Previous work associated WMH to HA [40,74]; thus, we chose the hippocampus as a region of interest (ROI). For the definition of the bilateral hippocampal masks (right and left), we used anatomical atlas-defined from Harvard Oxford Subcortical Structural Atlas implemented in FSL [75]. The region identified in the first step was subsequently used as a mask to perform ROI analysis using small-volume correction (SVC) procedure within the framework of the general linear model (GLM) in SPM8. We computed significant volume differences accounting for multiple comparisons through Family Wise Error correction (FWE) (*p* < 0.05) and a cluster extent threshold of 50 contiguous voxels. 

## 3. Results

### 3.1. Neuropsychological and Neuropsychiatric Measures

The two groups did not differ in terms of age, education, and general cognition (see Table 1).

As for neuropsychological variables, the DROCF score and the time (in sec) spent to complete the TMT part B were significant predictors for the presence of WMH (see Table 2). After the tolerance value estimation, they were both selected as independent variables in the multivariable logistic regression model. However, in the latter analysis interactions terms between the two predictors and their natural logs were significant [DROCF*ln(DROCF) Wald_1df_ = 4.27 p = 0.039; TMT-B*ln(TMT-B) Wald_1df_ = 5.50 p = 0.019) implying that assumptions for conducting the test were violated. 

Regarding neuropsychiatric variables, the BDI, ARS, STAI-Y1, STAI-Y2, STAXI-T, and FRS scores were significant predictors for the presence of WMH (see Table 2) in univariate binomial logistic regression models. After the tolerance value estimation, all variables were included in a multivariable logistic regression model that was significant (see Table 3) and explained 26% of total variance (adjusted R^2^) with a classification accuracy equal to 71.93% (73.33% for WMH+ and 70.37% for WMH−). The ARS and STAI-Y1 scores were significant predictors of WMH+ (see Table 3), while no interaction term between each continuous independent variable and its natural logarithm was significant (≥0.05), implying that the linearity assumption was not violated.

### 3.2. Regional Distribution and Volume Measurement of White Matter Hyperintensities (WMH)

**Figure 1:**
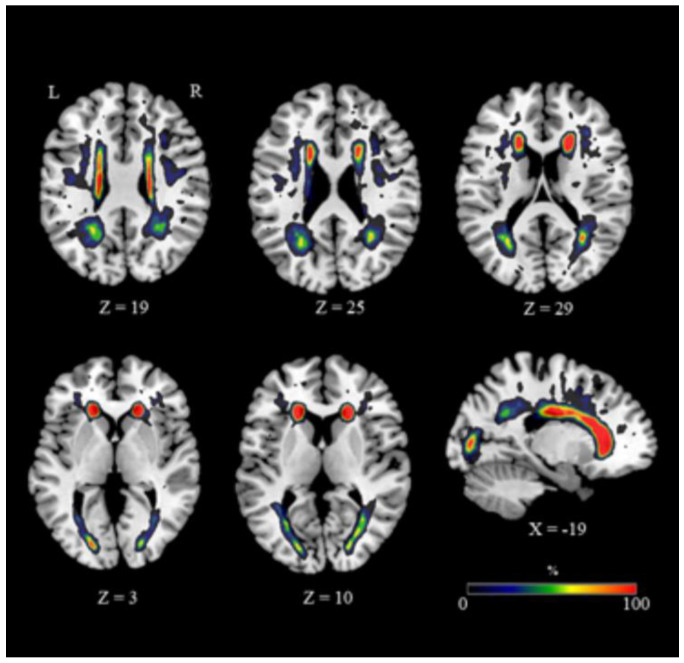
WMH distribution. Representative slices showing an overlapping of WMH maps. The color bar indicates the percentage of subjects overlapping obtained for each voxel. Legend: R, right; L, left; Coordinates are in Montreal Neurological Institute (MNI) space.

The extent and distribution of WMH varied considerably (Figure 1; mean volume ± SD = 7.869 ± 9.096 mm^3^, range 919–36.539) (Table 4). Anatomical WMH overlap maps were superimposed on the ICBM-DTI-81 white matter labels atlas [76]. Most of the altered voxels, indicative of undergoing damage, were localized in frontal areas, the anterior and superior corona radiata and the genu and body of the corpus callosum (CC) (Figure 1). More posterior regions were minimally affected by WMH (Figure 1).

### 3.3. Hippocampus–ROI Analyses

**Figure 2:**
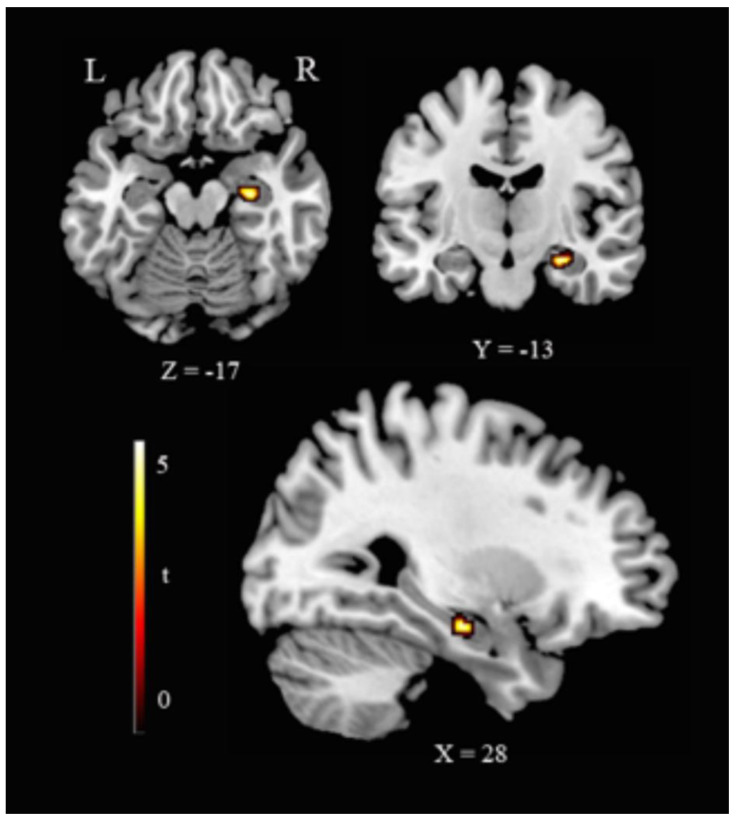
Hippocampal volume reduction in HC(WMH+). Representative slices showing VBM ROI-based results as for the contrast HC(WMH−) > HC(WMH+). Legend: R, right; L, left; Coordinates are in Montreal Neurological Institute (MNI) space.

WMH+ showed lower right hippocampus volume (cluster size 81 voxels, MNI coordinates: x = 28, y = −13, z = −17; t = 4.12, equivalent Z = 3.84, p (FWE corrected) = 0.019) (Figure 2).

## 4. Discussion

In this study, we investigated the association between WMH, subclinical cognitive, and neuropsychiatric phenomenology, and HA in healthy individuals aged between 50 and 80. We found that WMH+ had worse performance in executive functions and long-term visuospatial memory, higher subclinical depression, anxiety and apathy, a greater predisposition and hyperactivity to anger and increased fatigability. However, a multivariable analysis indicated that only subclinical anxiety and apathy were significantly associated with WMH. Finally, we found that WMH+ had lower right hippocampal volume.

The association of WMH with cognition, subclinical neuropsychiatric phenomenology, and HA has been separately explored in previous reports; however, our study is one of the few investigating such a relationship using a comprehensive approach.

### 4.1. WMH Are Associated with Decreased Cognitive Functioning

Despite the extensive research, the phenomenology associated with WMH in non-demented elderly subjects is still controversial. Previous neuroimaging studies, conducted with healthy elderly people free from dementia or mild cognitive impairment, established that WMH correlate with worse performance in specific cognitive domains including speed of information processing, executive functioning, and explicit memory [2,15,77]. Here, we found slightly significant differences in executive functioning (particularly rapid set-shifting) and long-term visuospatial memory between WMH− and WMH+ subjects.

It is well known that white matter injury reduces cognitive network efficiency. Indeed, neural networks underpinning cognitive functioning are widely distributed through the brain, and axons are the fundamental backbone of intraneuronal connections. Altered brain networks connection efficacy, due to white matter damage in aging, is hypothesized to be the substrate of subtle cognitive changes in seniors without dementia (O’Sullivan et al., 2001; Sullivan et al., 2001). In this context, our data strengthen the role of WMH as a proxy indicator of early damaged cortical connections that are critical for maintaining higher-order cognition [78], before overt cognitive decline occurs. Our findings also suggest that the link between WMH and subtle preclinical signs of cognitive decline may be detectable years before clinical symptoms of dementia typically emerge. Longitudinal studies demonstrated that WMH tend to develop from existing alterations and that higher baseline lesions lead to quicker WMH accumulation and faster cognitive decline in the elderly [79]. Thus, our study suggests that WMH might be considered a proxy biomarker for identifying individuals in mid and late life who are at greater risk for future clinically significant cognitive decline or dementia.

However, such findings emerged in the univariate analyses only and, as such, results should be taken with great caution. Indeed, it seems likely that the cognitive differences observed in the present study could be a byproduct of WMH and not primarily linked to the pathophysiological processes involved in WMH emergence.

### 4.2. WMH Are Associated with Subclinical Neuropsychiatric Changes

When neuropsychiatric symptoms were considered, we observed that the presence of WMH was associated with subclinical manifestations of depression, apathy, anxiety, anger, and fatigue. These findings are in line with the results of several studies suggesting subclinical neuropsychiatric symptoms as common occurrence in subjects showing variable degrees of WMH load [20,21,32,80,81,82] located mostly in frontal areas. Intriguingly, independently from WMH, a relationship between neuropsychiatric symptoms and WM structural changes was described in patents with mood disorders [83] and in healthy individuals free from mental disorders [84], thus strengthening the concept that damage in brain subcortical circuits (mainly streaming frontal areas) may be responsible for the emergence of neuropsychiatric symptoms, even at a subthreshold level.

The most remarkable finding of the present study is that, when a multivariable statistical approach was employed, only subclinical apathy and state anxiety were reliable predictors of WMH. Apathy is a common clinical feature of many neurodegenerative disorders, such as Alzheimer’s disease, Parkinson’s disease, progressive supranuclear palsy [33,85,86,87,88,89,90], and neurovascular disorders such as stroke [91]. The clinical expression of apathy is characterized by a significant reduction of goal-directed behavior, goal-directed cognition, and the emotional aspects of goal-directed behavior [55,92]. Apathy phenomenology ranges from subclinical apathetic-like status to severe conditions [42,93]. Although apathy has been extensively studied in relation to neuropsychiatric disorders, it is still unclear whether, in healthy people, its subclinical manifestation should be considered as a physiological phenomenon or whether it is a risk factor for progression to clinical disorders. A recent longitudinal study in non-demented older adults indicated that apathy is associated with increased risk of developing slow gait, frailty, and disability, independently from other established risk factor [94]. In pathological states, it has been suggested that apathy at baseline predicts which patients with amnestic-mild cognitive impairment will progress to AD [87]. Thus, apathy should be considered a mixed cognitive/neuropsychiatric disturbance related to ongoing AD neurodegeneration.

As for the cerebral hubs implicated in apathy, there is evidence of involvement of different, but closely interconnected, sub-regions of the prefrontal cortex and basal ganglia [95,96,97]. A common feature of all the pathological conditions in which apathy occurs is the presence of WM dysfunctions or lesions in the cortico-subcortical pathways that connect brain regions playing an important role in the regulation of emotions [98]. A fairly recent study on healthy individuals [42] also described the association between the occurrence of subclinical apathy and the presence of microstructural changes of WM regions such as the anterior thalamic radiation, the forceps major, and the corona radiata. The study suggested that these changes along with the alterations of the related white matter tracts, occur within a prefrontal-subcortical circuit leading to the emergence of apathy.

Pathological studies demonstrated that WMH are related to different severity of myelin degradation, loss of axons and oligodendroglia, gliosis, disruption of the ependymal lining, and/or infarction [99]. WMH are also associated with reduced blood perfusion in brain areas without WMH such as the basal ganglia and thalamus [100]. In line with the hypothesis by Mega and Cummings [101], our findings indicate that apathy might result from frontal-subcortical circuit changes that in healthy people intervene in complex cognitive functions and motivation, i.e., deep white matter afferents and efferents to the basal ganglia underlying reward processing, leading to the inaccurate perception or valuation of stimuli important for decision-making [102].

Our results indicate the association between WMH and subclinical state anxiety, which is a transitory emotional state that varies in intensity and fluctuates over time [57]. Several neuroimaging studies have attempted to identify the brain changes associated with mood and anxiety disorders. These studies were based on the hypothesis that state anxiety is related to dysfunctions regionally restricted to selected cortical and subcortical brain areas, such as the medial and caudo-lateral orbital cortex, the amygdala, the hippocampus, the ventromedial parts of the basal ganglia, and the ventromedial prefrontal cortex [103]. Further, previous Diffusion Tensor Imaging (DTI) studies [104,105,106] found that WM abnormalities, occurring in pathways linking the amygdala and other limbic regions to the ventromedial prefrontal cortex, may be the structural substrate of anxiety disorders.

Pieces of evidence converge on two potential brain areas involved in mechanisms of anxiety disorders [107]: (1) the hippocampus is involved in contextual information processing; fear response is influenced by information regarding safe versus potentially dangerous contexts. As such, hippocampal dysfunction has been implicated in the development of pathological anxiety, as a consequence of maladaptive appreciation for the contextual specificity of potentially dangerous stimuli; (2) animal studies suggest that medial prefrontal cortex damage interfere with normal extinction [108]. Altered extinction may thus lead to pathological anxiety; individuals with such deficits would be unable to capably modify previously experienced associations between harmless cues and dangerous stimuli, thus developing anxiety symptoms. We did not directly investigate this issue, but the indirect evidence that WMH were predominantly located in the frontal region of the brain and were associated to HA and subclinical anxiety is in line with previous findings. Future studies should be designed to clarify definitively this hypothesis.

Finally, it should be noted that, while univariate analyses indicated that subjects with WMH showed higher levels of depression, trait anxiety, and fatigue, such variables did not emerge as significant predictors of WMH presence in a multivariable model. It is important to state that based on this evidence alone, we are not suggesting that people with WMH do not suffer from such symptoms. Rather, these findings may suggest they are, to some extent, secondary to motivational loss, which is the more direct consequence of white matter damage, as suggested in previous studies (e.g., [33]).

### 4.3. WMH Are Associated with Hippocampal Atrophy

Pieces of evidence indicate that several brain changes, like WMH and cortical atrophy, co-exist in older adults [40] and contribute to impaired cognition [109]. However, few studies to date evaluated the causal effect of WMH on GM volume changes in elderly people free from cognitive impairment. In the present study, we found HA in healthy people with WMH. Many different mechanisms may explain our results. WMH can be considered as an index of cerebrovascular disease, and the relationship between WMH and HA suggests the importance of vascular pathology and hypoxia- and ischemia-driven insults [35]. Likewise, WM changes are mostly due to pathological alterations of the arteriole supplying the WM [110] and to the consequent loss of vascular integrity and dysfunction (or damage) of the blood-brain-barrier [111], thereby indicating a common vascular cause for WM and HA. Moreover, the co-existence of WMH and HA is associated with cerebral amyloid angiopathy (CAA), a feature also found in healthy aging individuals and leading to cognitive impairment [112]. Alternatively, WMH may contribute to HA through cortical disconnection [113] and/or WMH in tracts serving the hippocampus may lead to axonal loss and subsequent atrophy via Wallerian degeneration [40]. Thus, the HA observed in the present study might be a consequence of vascular insult determining both HA and WMH, or a downstream process subsequent to WMH via Wallerian degeneration.

## 5. Limitations

Before the conclusions, we would like to discuss some issues. First, our study has a cross-sectional design, and we could not analyze the temporal dynamics of the association between WMH and cognitive/neuropsychiatric phenomenology. Future studies need to investigate the causative role of WMH in the emergence of neuropsychiatric and cognitive phenomenology and HA through a longitudinal design, also in the perspective of the subclinical manifestation as prodromal stage of disease. Second, we used unimodal MRI analyses. It is possible that WMH lead also to WM microstructural alterations and functional dysfunctions that, in the present work, have not been taken into account. Future studies should consider the possibility of analyzing DTI and fMRI data in subjects with WMH and the putative associations with cognitive/neuropsychiatric measures.

## 6. Conclusions

Here, we highlight the role of WMH in the emergence of subtle and subthreshold cognitive and neuropsychiatric symptoms, as well as HA in healthy subjects. Our results are of possible clinical value. Indeed, cognitive changes, neuropsychiatric phenomenology, and HA are all risk factors of developing neurodegenerative diseases. From a preventive perspective, our results suggest that early interventions targeted to slow down the progression of WMH in midlife may complement interventions in the elderly aimed at preventing the development of cognitive impairment and dementia. In this view, WMH may be especially suitable to select subjects showing the highest risk for the development of cognitive decline and who may most benefit from primary prevention. The possibility of reducing the deleterious clinical effects of WMH should encourage greater efforts to prevent vascular contributions to cognitive impairment and dementia.

## Figures and Tables

**Table 1:** Demographic and cognitive characteristics of the 60 healthy subjects.

	HC (WMH−) (*n* = 30)	HC (WMH+) (*n* = 30)			
Variables	Mean (SD)	Mean (SD)	t	df	P
Age	64 (7)	64.6 (7)	−0.37	58	0.7
Education	13.8 (3.1)	12.23 (3.3)	1.79	58	0.07
Mini Mental State Examination (MMSE)	29.23 (0.89)	29.10 (1.21)	0.48	58	0.63

Legend: WMH+ thirty healthy subjects with WMH; WMH− thirty individuals without; df degrees of freedom; SD Standard deviation.

**Table 2:** Results from the separate binomial logistic regressions on group membership (WMH+ WMH−) and each neuropsychological and neuropsychiatric measure.

	Model		Predictor		
Neuropsychological Variables	χ^2^_(1df)_	*p* Value	OR	95% CI	*p* Value
MDB Rey’s 15-word Immediate Recall (RIR)	1.12	0.29	0.97	0.92–1.03	0.30
MDB Rey’s 15-word Delayed Recall (RDR)	0.39	0.53	0.94	0.78–1.14	0.53
Copy of Rey–Osterrieth Complex Figure Test (RROF)	3.34	0.07	0.85	0.71–1.02	0.09
**Recall of Rey–Osterrieth Complex Figure Test (RROF)**	**4.29**	**0.04**	**0.90**	**0.81**–**1.0**	**0.047**
Stroop test-word reading (ST_wr_)-time (s)	2.01	0.15	1.18	0.93–1.50	0.17
Stroop test-color naming (ST_cn_)-time (s)	4.53	0.03	1.17	1.0–1.36	0.05
Stroop test-interference (ST_i_)-time (s)	1.80	0.18	1.04	0.98–1.11	0.20
Raven’s Progressive Matrices ‘47	3.77	0.05	0.90	0.80–1.0	0.06
Copying Drawings	2.23	0.13	0.69	0.41–1.14	0.14
Copying Drawings with Landmarks	3.94	0.05	0.87	0.74–1.01	0.07
Double Barrage Test (DBT)-time (s)	2.21	0.14	1.02	1.0–1.05	0.17
Double Barrage Test (DBT)-Recognition	0.67	0.41	1.14	0.82–1.58	0.42
Double Barrage Test (DBT)-False	0.06	0.80	1.13	0.43–2.93	0.80
Trail Making Test (TMT A)-time (s)	2.63	0.10	1.02	1.0–1.06	0.14
**Trail Making Test (TMT B)-time (s)**	**6.94**	**0.001**	**1.02**	**1.0**–**1.04**	**0.04**
Phonological verbal fluency	0.58	0.45	0.98	0.94–1.03	0.45
Semantic verbal fluency	0.70	0.40	0.97	0.88–1.05	0.40
Wisconsin Card Sorting Test (WCST)-Pers Err	0.32	0.57	1.18	0.65–2.12	0.58
Wisconsin Card Sorting Test (WCST)-non Pers Err	1.68	0.19	1.46	0.8–2.68	0.21
**Neuropsychiatric variables**					
Hamilton Depression Rating Scale score	2.82	0.09	1.12	0.98–1.28	0.11
**Beck depression inventory (BDI) score**	**6.44**	**0.01**	**1.18**	**1.03**–**1.36**	**0.02**
**Apathy Rating Scale (ARS) score**	**6.40**	**0.01**	**1.18**	**1.03**–**1.35**	**0.01**
**State Anxiety Inventory Stai Y 1 score**	**10.66**	**0.001**	**1.18**	**1.05**–**1.31**	**0.003**
**Trait Anxiety Inventory Stai-Y 2 score**	**7.88**	**0.005**	**1.11**	**1.02**–**1.22**	**0.01**
Hamilton Anxiety Rating Scale (HAMA) score	1.24	0.27	1.06	0.95–1.17	0.28
State-Trait Anger Expression Inventory (STAXI)-S score	1.07	0.30	1.91	0.39–9-48	0.43
**State-Trait Anger Expression Inventory (STAXI)-T score**	**4.76**	**0.03**	**1.17**	**1.0**–**1.35**	**0.04**
State-Trait Anger Expression Inventory (STAXI)-R score	4.05	0.04	1.07	1.0–1.14	0.06
**Fatigue Rating Scale score**	**7.65**	**0.005**	**1.24**	**1.04**–**1.48**	**0.02**

Legend: df degrees of freedom; CI confidence interval; OR odd ratio; Significant predictors are highlighted in bold.

**Table 3:** Results from the multivariable logistic regression on group membership (WMH+ WMH−) and significant neuropsychiatric predictors.

	Model		Predictor		
Neuropsychiatric Variables	χ^2^_(6df)_	*p* Value	AOR	95% CI	*p* Value
**Whole model fit**	**20.70**	**0.002**			
Beck depression inventory (BDI) score			0.99	0.79–1.26	0.98
**Apathy Rating Scale (ARS) score**			**1.20**	**0.99**–**1.44**	**0.04**
**State Anxiety Inventory Stai Y 1 score**			**1.51**	**1.0**–**1.32**	**0.04**
Trait Anxiety Inventory-Stai Y 2 score			0.98	0.85–1.12	0.73
State-Trait Anger Expression Inventory (STAXI)-T score			1.04	0.86–1.25	0.70
Fatigue Rating Scale score			1.24	0.97–1.59	0.09

Legend: df degrees of freedom; CI confidence interval; AOR adjusted odd ratio; Significant predictors are highlighted in bold.

**Table 4:** White matter hyperintensities volume (in mm^3^) and distribution in the WMH+ group.

Participant	Periventricular WMH Volume	Subcortical WMH Volume	Total WMH Volume
1	3012	281	3293
2	5853	1783	7636
3	4453	1236	5689
4	2237	172	2409
5	1483	275	1758
6	84	835	919
7	2008	410	2418
8	6317	5329	11646
9	2014	198	2212
10	920	272	1192
11	1187	4708	5895
12	1878	528	2406
13	939	1055	1994
14	8801	22,727	31,528
15	1287	1273	2560
16	4895	1301	6196
17	13362	16,489	29,851
18	2445	674	3119
19	15436	21,103	36,539
20	1621	541	2162
21	3973	1234	5207
22	5643	2537	8180
23	4493	1513	6006
24	7378	6072	13450
25	2022	317	2339
26	6421	3796	10217
27	8271	3815	12,086
28	2990	1324	4314
29	5248	2967	8215
30	3156	1507	4663
